# On the Removal of Naso-Pharyngeal Tumors

**Published:** 1884-11

**Authors:** W. Thornley Stoker


### On the Removal of Naso-Pharyngeal Tumors.

BY W. THORNLEY STOKER, M.D., F.R.C.S.

The term "naso-pharyngeal" has been applied in such different senses to tumors and polypi involving the nasal and pharyngeal cavities, that I may preface the remarks I have to make by saying that I use the expression only in reference to growths which, while invading the naso-pharyngeal space, have their origin in the osseous tissue forming its roof, usually the body of the sphenoid, or the basilar processes of the occipital bone, which, in fact, grow from the base of the skull. Although these masses form numerous secondary attachments, and although it is for that reason often difficult to distinguish their primary seat, yet it is generally possible to do so, and observers are sufficiently well agreed that they almost invariably spring from the situation I have mentioned.

The constitution of the naso-pharyngeal growths is various—most frequently, perhaps, of the fibrous, sarcomatous or cartilaginous orders, or some derivative or admixture of them. They are not infrequently malignant, and are very likely to be rapid and destructive in their growth.

As regards shape, there are two varieties—sessile and pedunculated, the latter forming the so-called naso-pharyngeal "polypi," while to the former the term "tumors" is more properly applied. With the polypi or pedunculated growths I have no concern in my present communication. They are, as a rule, fibrous in structure, comparatively innocent, and easy of removal, not requiring any formidable operation, unless it is found impracticable to surround the pedicle with a noose, or to divide it through the nose with a cutting instrument. My remarks have to do with those growths which are generally of a more virulent nature, and whose broad attachments require severe operations for their exposure and division.

I need not dilate upon the horrible consequences produced by an extending naso-pharyngeal tumor to excuse myself for bringing forward any cases which can throw a light upon the surgery of this condition. We have all watched the terrible progress of these tumors when unchecked by operation. We have seen them extending in different directions, and successively destroying the senses of smell, hearing, and sight, destroying their victims by exhaustion, the result of discharge, hemorrhage, want of sleep, or dysphagia, suffocating him by extension toward the larynx, or, worst of all, after he has passed through an Inferno of suffering, invading his brain and leaving him toward his end in the dreadful condition of a creature without senses, possessing no functions save respiration, digestion, and circulation, with little life beyond that of a vegetable, an object of horror to all around him.

For these broad based growths, if they are in a state to admit of operation, there is but one measure that should be attempted —complete and thorough ablation. The great difficulty of this is found in the remoteness of the attachments, and the danger in exposing them sufficiently to insure their complete extirpation. Once the connections of the tumor have been thoroughly brought to command, it matters little by what means they are divided— the surgeon may please himself as to scoop or knife or cautery. The point which causes him the most anxiety is the primary stage of the operation, the proper exposure of the tumor so he can reach its attachments.

Three classes of operations have been practiced for this purpose:

1. Through the palate.
2. By partial or complete removal of the upper jaw.
3. By displacement of the upper jaw.

Of the operations through the palate, the most reputable, perhaps, is that of Nelaton, but bad is the best, because it not only affords a very difficult and limited access to the naso-pharangeal cavity, but it exposes the part of the tumor most remote from its attachments.

The second class of operations, partial or complete removal of the jaw as a means of exposing the disease, was first recommended by Whately, and practiced by Syme. It may still be occasionally called for, but most surgeons will probably regard it as obliterated from the list of usual procedures by the operations of the third class, those of displacement. In these latter, one of which should, if possible, be selected, the upper jaw, on one or both sides, is, by a complicated operation, divided superiorly, separated from its attachments, and displaced downward and outward. This method of operating has been practiced with success by Huguier and Langenbeck, and has the advantage over any ablation of the jaw that the parts are replaced when the disease is removed, no deformity remains, and the dental arch is restored.

My observation of the naso-pharyngeal growths have led me to the belief that in the great majority of cases such heroic measures are unnecessary, and that the disease can be attacked and removed through more limited openings than the measures I have named are intended to afford. I have two reasons for coming to this conclusion: First, that the growth of the tumor has, by the time the operation is demanded, separated the superior maxillæ to a considerable extent, so that as the surgeon cuts or hacks away the disease, he finds his fingers in a cavity where he has room enough for action and exploration; and second, that the rough kind of cutting, scooping, and gouging which is the best way of attacking these tumors, does not require much space for its exercise.

I do not wish to be understood as denying the necessity of the more formidable operation in some cases. What I seek to convey is that cases which to all appearances require these extreme measures, may be radically dealt with by minor means, and I do not think that a surgeon can ever say with certainty what steps he may have to take until he begins to operate on one of these cases. * * * *

The points, as I have said, which my cases seem to teach, are:

1. That, for reasons which I have already given, such an operation as those described generally afford sufficient access and room without having recourse to more heroic measures, and,

2. That, having the foregoing in view, there is hope from even the most unpromising instances of the disease.—*Medical Press.*

